# Anticipating Climatic Variability: The Potential of Ecological Calendars

**DOI:** 10.1007/s10745-018-9970-5

**Published:** 2018-02-16

**Authors:** Karim-Aly S. Kassam, Morgan L. Ruelle, Cyrus Samimi, Antonio Trabucco, Jianchu Xu

**Affiliations:** 1 Department of Natural Resources and the American Indian and Indigenous Studies Program, Cornell University, Ithaca, NY USA; 2 Department of Geography, University of Bayreuth, Bayreuth, Germany; 3 Impacts on Agriculture, Forests and Ecosystem Services Division, Euro-Mediterranean Center on Climate Change, Sassari, Italy; 4 Center for Mountain Ecosystem Studies, Kunming Institute of Botany, Chinese Academy of Sciences, Beijing, China

## Introduction: The Issue

Indigenous and rural societies who have contributed least to anthropogenic climate change are facing its harshest consequences. One of the greatest challenges of climate change is lack of predictability, especially at the local scale. An estimated 70-80% of the world’s food is produced by small-holders with less than two hectares of land (FAO 2014; Lowder *et al.* 2016). These small-scale farmers and herders face an ever-shifting ‘new normal’ climate, increasing inconsistency in the seasonality of temperature and precipitation, and higher frequency of what were once considered extreme weather events (Jolly *et al.* 2002; Thornton *et al*. 2014). Climate variability is disrupting food systems and generating a debilitating anxiety (Carroll *et al*. 2009; Kassam 2009a,b; Coyle and Susteren 2011; UN Human Rights Council 2016). Anticipatory capacity – the ability to envision possible futures and develop a plan of action to deal with uncertainties – is needed urgently (Tschakert and Dietrich 2010).

Communities and researchers must create innovative systems to recognize and respond to climate trends and prepare for a greater range of possible scenarios (Reid *et al*. 2014; Cuerrier *et al*. 2015). To build anticipatory capacity for climate change, communities need systems that are effective at the scale of the village and valley (Berkes and Jolly 2001; Downing and Cuerrier 2011). While climate scientists have increased model capabilities to make more accurate projections of global climate conditions, the uncertainties of global climate modeling together with those of downscaling methods means that these models are not always reliable at regional and local scales (Salick and Ross 2009). Synergy between indigenous ecological knowledge and climate science has already benefitted many local communities, as well as international understanding of climate change drivers and impacts (Jolly *et al*. 2002; Nickels *et al*. 2005; Nyong *et al.* 2007; Kassam 2009a; Alexander *et al.* 2011; Boillat and Berkes 2013; Rapinski *et al*. 2017;). Similarly, ground-truthing climate models with indigenous ecological knowledge can be used to refine downscaling methods and to inform planning and policies at local, regional, and national levels.

Projections of climate models are least accurate within mountainous regions, where weather stations are scarce and rugged topographies dramatically alter climate patterns (Hall 2014). In addition, significant environmental degradation in many mountain regions, such as reduction of vegetation cover due to overgrazing or hydrological transformations resulting from road and dam construction, are obscuring the entangled effects of climate change. Nevertheless, food producers in these remote regions require the ability to anticipate patterns of temperature, precipitation, and runoff from glaciers and snowfields.

Many indigenous and rural societies have developed unique systems to recognize and respond to climatic trends and variability. Over the course of multiple generations living in particular landscapes, indigenous people have accumulated knowledge of the relative timing of celestial, meteorological, and ecological phenomena. Understanding these relationships has enabled these communities to anticipate weather and other seasonal processes, and thereby coordinate their livelihood activities (Acharya 2011; Turner and Singh 2011). However, indigenous knowledge systems have suffered centuries of disruption and destruction as a result of colonialism, violent conflicts, and loss of land. Global climate change introduces unprecedented rates and magnitudes of change, exacerbating existing inequities (Turner and Clifton 2009). Although inadequately investigated, evidence suggests that climate change has impacted the physical, mental, and emotional health of indigenous peoples (Cunsolo Willox *et al*. 2012, 2014).

In this brief communication we suggest a new approach for applied participatory action research to build anticipatory capacity for climate change. Specifically, we describe the development of ecological calendars that integrate indigenous knowledge and scientific data, and therefore require input from both *communities of inquirers* and *communities of practice*. We provide a case study of our ongoing work in the Pamir Mountains of Afghanistan, China, Kyrgyzstan, and Tajikistan, where we are in the midst of transdisciplinary research with indigenous agropastoralists.

## Re-Framing: The Role of Time

Developing anticipatory capacity for anthropogenic climate change requires a re-conceptualization of the notion of time. In industrialized societies, time is thought of as a metronomic progression represented by the familiar Gregorian calendar. However, we know that our experience of time is context-specific and therefore unique because it is embedded in socio-cultural meaning. Furthermore the timing of ecological processes and events are not consistent from year to year, and as a result of climate change, they increasingly vary, taking place at different dates on the Gregorian calendar. At our study sites in the Pamir Mountains of Central Asia, ice break-up, thawing, ploughing, sowing, and harvesting begin between 15 and 40 days earlier than a decade ago. In order to make sense of this variability, it may help to think of time as relational. For instance, our research from Lake Oneida in the Northeast United States indicates that historically, the blossoming of a certain flower indicates that the ground has thawed, ploughing can begin, burial services can be performed for those who have died during the winter, a particular fish is running in the river, and traps can be set for small animals that have emerged from hibernation. Knowledge of temporal relations allows communities to synchronize their livelihood activities within their ecological context. Re-conceiving time as relational may help us anticipate climatic variation, enabling us to coordinate our activities with variable biophysical phenomena even as their timing within the Gregorian calendar becomes less predictable.

A re-conceptualization of time as unique, flexible, and relational underlies the notion of an ecological calendar: a dynamic, experiential framework to understand time. Ecological calendars (also known as natural or phenological calendars) are knowledge systems to measure and give meaning to time based on close observation of one’s habitat. They are comprised of seasonal indicators that include abiotic phenomena, such as the first snowfall or last frost, as well as biotic events, such as the flowering of a certain tree or the arrival of a migratory bird species. These calendars differ from celestial calendars, such as the familiar Gregorian calendar, in that they do not rely solely on fixed cycles of the sun, moon, or stars. Unlike those cycles, the indicators within an ecological calendar respond to climate and other seasonal processes that directly impact livelihood activities. By referring to seasonal cues, the measurement of time becomes flexible with respect to celestial cycles, and communities can identify the optimal timing for their activities. Therefore, ecological calendars may enhance anticipatory capacity for climate change by enabling communities to synchronize their activities with their ecosystem to accommodate climate trends and increasing variability.

Ecological calendars are not new; they have been documented among indigenous peoples inhabiting a diversity of ecosystems, from the mountains of Central Asia (Kassam *et al.* 2011) to coastlines and deserts of Oceania (Mondragón 2004; Prober *et al.* 2011) to the rainforests of the Amazon (Cochran *et al*. 2016). Traditional ecological calendars are based on context-specific phenological knowledge generated by communities that have inhabited particular landscapes for multiple generations. Therefore, each of these calendars is unique because it is embedded in the relationships of people to their own ecosystem. For example, First Nations in western Canada recognize reliable synchronies between the singing of birds and the ripening of edible berries (Lantz and Turner 2003). In Vanuatu, the call of a sandpiper cues the harvest of a sea worm at the next full moon (Mondragón 2004). Communities constantly adapt their ecological calendars to remain relevant to changing socio-cultural and ecological conditions. Through continuous application, these calendars are inherently dynamic, giving them vibrancy, relevance and longevity.

Unfortunately, many ecological calendars have been suppressed or destroyed by colonial powers. Deliberate efforts to colonize not only the lands but also the minds of indigenous peoples have undermined their knowledge systems, including traditional calendars (Kassam *et al.* 2017). Now, many indigenous communities are looking to revitalize their ecological calendars because of the pragmatic necessity of building anticipatory capacity for climate change (Kassam *et al.* 2011; Prober *et al.* 2011; Woodward *et al*. 2012; Turner and Spalding 2013).

At the same time, several European nations have begun to develop new ecological calendars, using statistical analyses of historical records to identify correlations between phenological cues and relevant climate variables (Ahas *et al.* 2000; Ahas and Aasa 2003; Morisette *et al*. 2009). For example, Estonian researchers analyzed farmers’ diaries and identified a sequence of 24 plant phenological phases that are reliably correlated with air temperature and therefore may be used to guide agricultural activities under a more variable climate (Ahas *et al.* 2000). As we demonstrate, both the revitalization of traditional and development of new ecological calendars benefit from a combination of indigenous and scientific knowledge and therefore require close collaboration among diverse knowledge holders.

## Case Study: Ecological Calendars in the Pamir Mountains

The Pamir Mountains of Central Asia are a crossroads of time, culture, and ecosystems. Agropastoralist communities in these mountains developed complex ecological calendars known as ‘calendars of the human body,’ which included two periods of counting days on the human body. In the spring, a *hisobdon* (‘keeper of time’) closely observed seasonal phenomena before initiating the first period of counting, beginning at the toe and proceeding to the head. After a period without counting in mid-summer, counting began again at the head and moved back toward the toe. These ecological calendars were highly diverse, adapted to the specific valleys and villages where they were used to guide farming, herding, and hunting activities.

Calendars of the human body reflect an intimate knowledge of the complex connectivity between the human body, agricultural activities, and ecological processes. Analysis of historical manuscripts indicate that these calendars have been in use for at least 600 years, although they are likely much older (Kassam, *et al.* 2011). In the first half of the twentieth century, ethnographers documented calendars of the human body among the diverse ethnic groups along the Panj River and its tributaries in Afghanistan and Tajikistan (Bobrinsky 1908; Lentz 1939; Andreev 1958; Kislyakov and Pisarchik 1966;). We have documented additional calendars, expanding their geographical range and the number of ethnic groups known to have used them (Kassam *et al.* 2011). It is likely that calendars of the human body or similar ecological calendars have been used in other parts of the Pamir, including in Kyrgyzstan and China.

During field research in the Pamirs in 2006 and 2007, discussion of the ecological calendars arose as a result of dramatic climatic variation. As in other mountain regions (Beniston and Stoffel 2014), livelihoods are directly linked to water provided from glacier melt, snow cover, and precipitation. For example, the temporal and spatial distribution of snow cover is a main factor determining the seasonal use of pastures. Pamiri villagers report that calendars of the human body enabled their ancestors to synchronize their livelihood activities with seasonal changes. However, under the command economy of the Soviet Union, these ecological calendars were suppressed and fell out of use. With the fall of the Soviet Union and increasing anthropogenic climate change, villagers expressed a strong desire to revitalize and recalibrate their ecological calendars as a source of anticipatory capacity for food security.

**Fig. 1:**
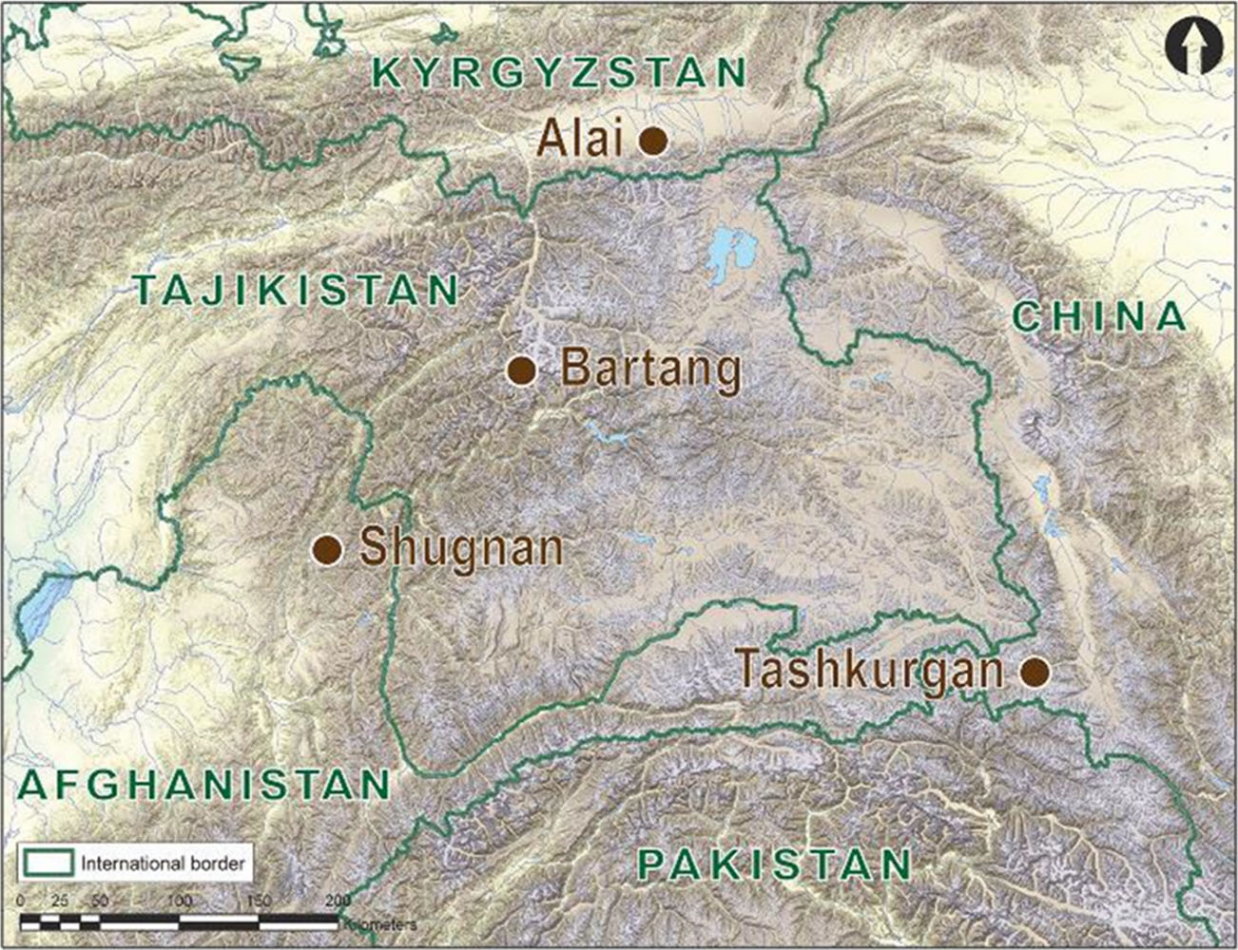
Study sites for transdisciplinary research on ecological calendars in the pamir mountains

In 2015, an international team of scientists from the USA, China, Germany, and Italy began to conduct participatory action research on ecological calendars in the Pamir (Fig.1). The goal of the project is to use scientific data and indigenous knowledge to revitalize Pamiris’ traditional calendars as a source of anticipatory capacity for climate change.

## Insights from Research Process

### Transdisciplinary Collaboration

**Fig. 2:**
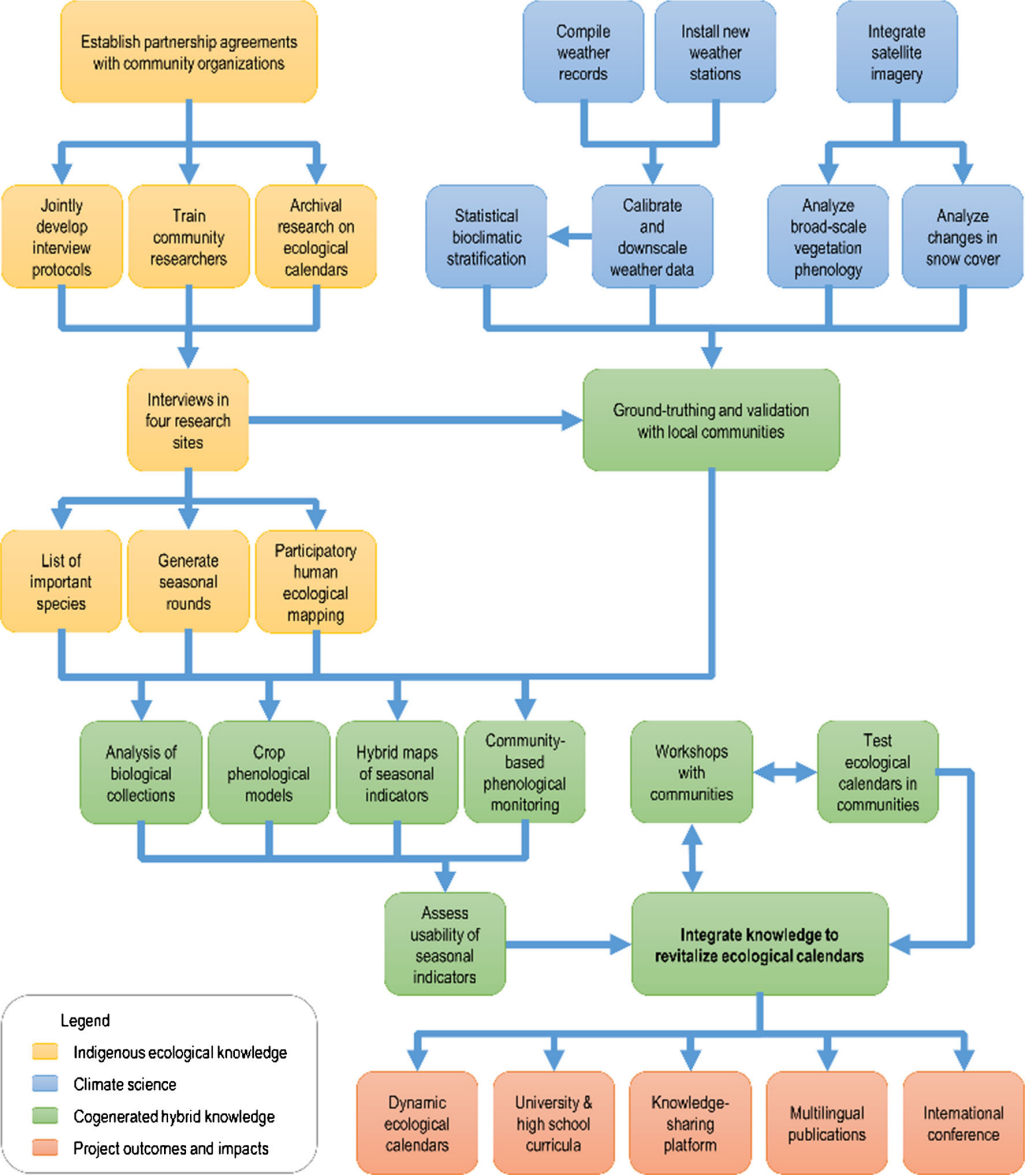
Transdisciplinary Research Process and Knowledge Integration; blue boxes represent the measuring and modeling of biophysical data; yellow boxes indicate the documentation and analysis of indigenous ecological knowledge; both streams of knowledge are used to undertake the activities shown in green boxes, including iterative validation and cogeneration of results; and red boxes show how results will be disseminated locally and then internationally

Revitalization of ecological calendars is only possible through transdisciplinary collaboration. Because local communities are key ecological knowledge holders, their participation in all stages of research design and implementation is essential so that outcomes have immediate impact and maximum long-term benefit. Furthermore, it is important to incorporate scientific data on the status and dynamics of change. Data on climate change often have spatial and temporal scales much coarser than processes affecting livelihood activities; so one of the challenges for effective collaboration is integrating information across different scales (Fig. 2).

A transdisciplinary framework not only needs to bring together scientists and practitioners but also diverse expertise from within both groups. For example, among the *community of inquiry*, anthropologists, botanists, climatologists, ecologists, educationalists, and ethnographers may form an interdisciplinary team. Among a *community of practice*, beekeepers, farmers, fishers, gatherers, hunters, orchardists, pastoralists, and teachers contribute as participants in the cogeneration of hybrid knowledge by engaging in joint analysis of the scientific data with local knowledge.

Just as the *community of inquiry* is diverse, bringing to bear different scientific information that requires dialogue to achieve commensurability, equally the *community of practice* is marked by heterogeneity in indigenous knowledge, often related to ecological professions and socio-cultural context. For instance, the ethnically Kyrgyz villagers of Sary Mogul, in the Alai Valley, are characterized as herders. Whereas, the ethnically Bartangi villagers of Savnob and Roshorv, also in the Pamir Mountains, are largely farmers. Therefore, grain production in Sary Mogul is mainly for animal consumption, whilst in Savnob and Roshorv cereals are intended primarily for human needs. Hence, their diverse livelihoods affect their knowledge systems.

Inter-village heterogeneity is mirrored in intra-village difference. For example, among residents of Sary Mogul there are different banks of knowledge as a result of their settlement histories. Despite shared Kyrgyz origins, the settlement of villagers in Sary Mogul was influenced by the former Soviet Union and post-Soviet developments. Until the 1940s, there were no permanent settlements in the region, although Kyrgyz pastoralists used the area. Sary Mogul was founded in 1946 and was geographically within the Kyrgyz Soviet Socialist Republic. However, it was administratively under the control of the Tajik Soviet Socialist Republic effectively spurring resettlement of other Kyrgyz peoples from the Eastern Pamirs of Gorno-Badakhshan Autonomous Region (Sonntag 2016). Similarly, under the Soviet system, forced but short-lived resettlement of residents from the Bartang Valley to the lowlands of Tajikistan for cotton farming added to the disruption of ecological knowledge in Savnob and Roshorv (Kassam 2009b). This colonial history and process of settlement affects the knowledge systems of the peoples of the Pamir.

Furthermore, given the practice of marrying women from outside the village, knowledge seems to follow patrilineal structures. For example, women who have married into families from Roshorv and Savnob initially find themselves in a different ecological context. However, gendered difference in intra-village knowledge is not a lasting feature of a woman’s experience as she engages her new habitat. Moreover, she brings fresh understandings of agricultural practices from her own village of origin. As we have shown elsewhere, though these differences between and within villages present challenges, they also contribute to the robustness and resilience of knowledge systems (Ruelle and Kassam 2011). These differences are an adaptive asset, in spite of a tragic colonial history, for revitalizing ecological calendars.

### Generating the Seasonal Round

**Fig. 3:**
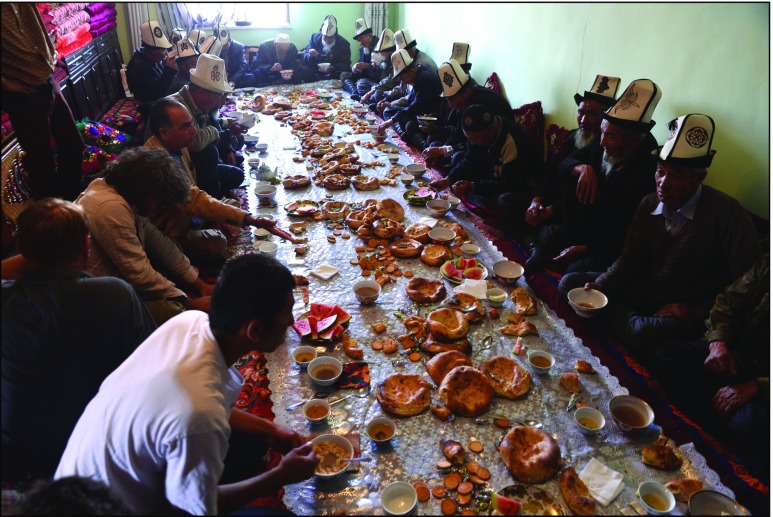
Meal with village elders in Sary Mogul prior to generating seasonal round

The first step in undertaking collaborative research on ecological calendars is to involve community members in the generation of illustrated seasonal rounds. This process provides insight into livelihood activities linking socio-cultural and ecological events. We, the *community of inquirers*, using local networks established by our community partners invite relevant participants, host a meal, explain our objectives, and then listen to knowledge holders. The meal is key in establishing a relationship and sharing ideas (Fig. 3).

**Fig. 4:**
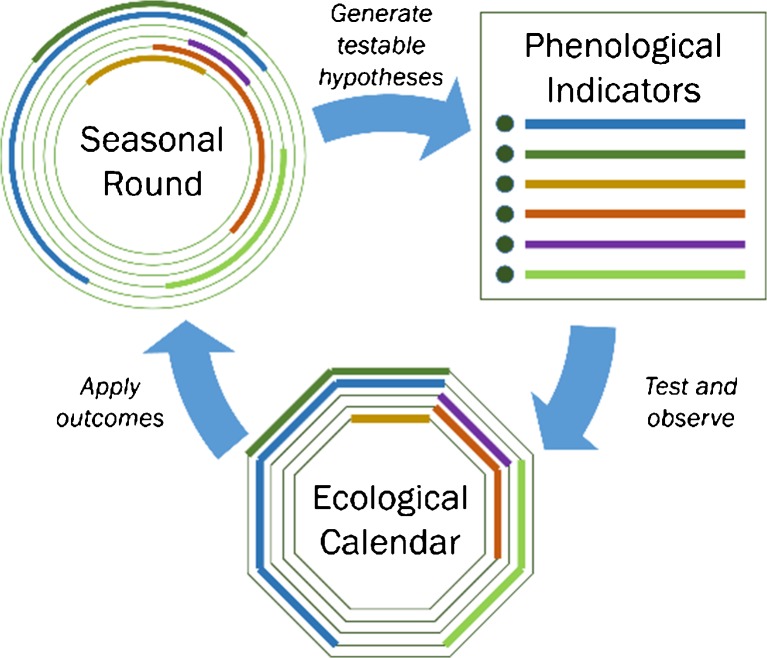
Iterative process of generating and adapting ecological calendars

After the meal, we begin with a simple question: “how do you know that winter has ended and spring has arrived?” This sets into motion a process of recounting seasonal events with key biophysical indicators (Fig. 4). At all the research sites we have found that by gathering *communities of practice* in a workshop setting where *inquirers* primarily listen to understand how the indigenous experience of time and system of time-keeping works, we are able to co-produce community specific seasonal rounds. A young member of the community facilitates the discussion and documentation on a diagram representing the cycle of one year.

Seasonal rounds are place-specific and their development includes diverse members of the community. Integrating various perceptions and concepts from different generations, economic status, educational levels, gender, and ecological professions is key. This framework has enormous advantage as it provides a climate service that supports informed decision-making tailored for subsistence communities.

### Identifying Reliable Seasonal Indicators

Seasonal indicators predate modern climate models and weather forecasts as the first climate services used by humanity to plan livelihood activities (Sah 1979; Hewitt *et al.* 2012). By developing seasonal rounds of agricultural and social activities, indicators are revealed and their connections are highlighted. Close attention is paid to the components of the food system, including fruits, vegetables, grains, legumes, animals (domesticated and hunted), insect pests, invasive species, and diseases. Developing consensus on the use of specific seasonal indicators is important to test and verify the reliability of phenological indicators to regulate activities affected by climate.

During discussions of the seasonal round, participants will generate a list of key species and biophysical phenomena for further investigation to determine if they are reliable indicators to include in ecological calendars. The decision to focus on certain indicators must be community-based in order to prioritize what is directly relevant. By selecting indicators of which communities are highly cognizant, the project team ensures that communities can later adapt their calendars to changing climatic conditions. Therefore, for a seasonal phenomenon to be included as an indicator within an ecological calendar, it must be: 1) known by community members, 2) observable throughout the landscape, 3) occurring in synchrony with livelihood activities, and 4) responsive to climate trends and variability. Interviews, mapping, and transect walks establish whether a seasonal indicator is widely known within the community, if it can be observed by a majority of community members, and how it may be identified and measured. As a result, selection of indicators by communities will enhance the adaptive capacity of ecological calendars and their long-term sustainability.

When we identify reliable seasonal indicators, we can test and observe the indicators in real-time to revitalize ecological calendars, thereby refining a tool for rural communities to maintain understanding of their habit and the changes within it. This is and will continue to be an iterative process where community members are not only observers of climatic events but also the key actors in developing their anticipatory capacity over the long run.

### Cogenerating Knowledge

A participatory approach enables the research team to document linkages between bioclimatic events and livelihood practices. It further enables the team to obtain on-the-ground, real-time understanding of agropastoral activities that are central to effectively functioning ecological calendars. During joint fieldwork, measurements from weather stations, climate data, and satellite imagery should be discussed with local communities to interpret this information in relation to their observations. Collaborative, transdisciplinary fieldwork ensures the interdisciplinary link of data on climate change and local knowledge. Hybrid knowledge resulting from the combined analysis of the scientific data (including historical weather data and multispectral remote sensing datasets) with human ecological mapping will provide a basis for community-based, scientifically informed selection of relevant seasonal indicators for ecological calendars. Indigenous and rural communities will be the primary beneficiaries and implementers of the research outcomes; namely, revitalized ecological calendars to anticipate climate change.

**Fig. 5:**
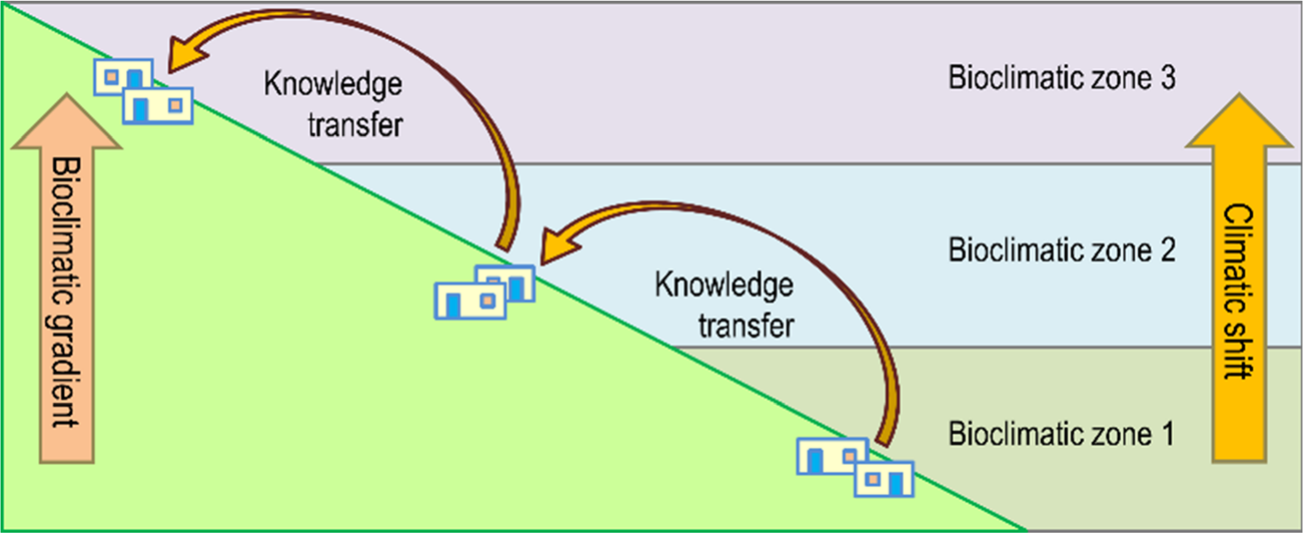
Transfer of hybrid knowledge along bioclimatic gradients

A participatory approach facilitates cogeneration of hybrid knowledge based on weather records, herbarium specimens, phenological data, satellite imagery, climate models, and indigenous knowledge to revitalize calendars for increasing climatic variation. For instance, analysis of seasonal rounds combined with crop modelling will determine whether the timing of seasonal indicators align with farming and herding activities. Moreover, such knowledge can be transferred to other communities along bioclimatic gradients to anticipate shifts in climate (Fig. 5). Revitalization and development of new ecological calendars is a promising, innovative approach for climate adaptation anywhere in the world and provides meaningful climate services.

## Impacts and Policy Implications

### Intellectual and Ethical Imperative

As noted at the outset, indigenous and rural communities that contributed least to the causes of anthropogenic climate change are now suffering its harshest impacts. Collaboration between *communities of inquirers* and *communities of practice* is therefore an ethical imperative. This is made more pertinent since the scientists and researchers who make up the *community of inquirers* have either benefited from or reside in societies that have caused anthropogenic climate change. Development of ecological calendars should be viewed as an opportunity to revitalize the fundamental relationship between people and their habitat, between diverse knowledge systems, and between indigenous rural communities and urban societies.

### Praxis

**Fig. 6:**
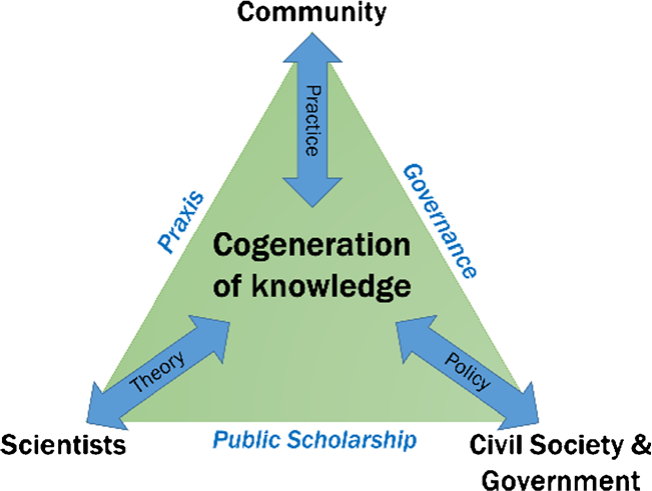
Cogeneration of knowledge in practice

Public scholarship in the form of applied research on ecological calendars requires collaboration with institutions that frame and implement policy. We have found that communities use indigenous ecological knowledge for climate change adaptation but it is not incorporated into policy formulation. Civil society organizations and government institutions have a key role to play in the participatory process. Their involvement at the conception of the research aids effective planning for impact and policy formulation once ecological calendars have been revitalized (Fig. 6). Links with civil society and governmental institutions is central to accessing resources, advice, and strategically implementing outcomes of our applied research. We have established partnerships with 13 governmental and civil society organizations globally to achieve maximum impact.

### Future Directions

At the core of this research is the policy objective of securing food and livelihood systems of mountain societies. The dynamic relationship among communities, researchers, and policy makers facilitates specific outcomes:

- Transfer of knowledge between communities in different bioclimatic zones through workshops so that shared insights inform praxis;
- Hosting an international conference focusing on ecological calendars to improve food security and resilience; and
- Development of school curricula for inter-generational transfer of knowledge and continued adaptation of calendars.

Effective responses to anthropogenic climate change necessitate public scholarship through respectful collaboration for cogeneration of knowledge among diverse scientific, community, civil society and governmental institutions.

Climate change exacerbates existing inequities such as poverty, food insecurity, social injustice, *et cetera*. Such structural problems defy singular policy formulations because their root causes are contingent, context-specific, and difficult to perceive. As a result, there are often conflicting understandings of the issues. Moreover, there is a possibility that variation in weather patterns in combination with existing inequities may have such quantum impacts, which are so rapid and volatile, that there are no effective means to anticipate change. While we must consider this possibility, it is not a determined outcome. Therefore, we have argued that effective responses to building anticipatory capacity need to be collaborative, because no one set of expertise is sufficient, and diversity of knowledge and experience is essential. A participatory approach to developing anticipatory capacity through complex engagement of socio-cultural and ecological systems is not only desirable but, more importantly, a necessity.
